# Anti-PD-1 antibody therapy for epithelial skin malignancies: An investigator-initiated, open-label, single-arm, multicenter, phase II clinical trial (NMSC-PD1 Study)

**DOI:** 10.1097/MD.0000000000022913

**Published:** 2020-10-30

**Authors:** Maki Ishii, Ikuko Hirai, Keiji Tanese, Takayuki Fusumae, Yoshio Nakamura, Keitaro Fukuda, Hiroshi Uchi, Kenji Kabashima, Atsushi Otsuka, Kenji Yokota, Naoya Yamazaki, Kenjiro Namikawa, Taku Fujimura, Tatsuya Takenouchi, Yuki Yamamoto, Mana Nishiguchi, Yasunori Sato, Masayuki Amagai, Takeru Funakoshi

**Affiliations:** a Department of Dermatology, Keio University School of Medicine, Tokyo; b Department of Dermato-oncology, National Hospital Organization Kyushu Cancer Center; c Department of Dermatology, Kyoto University Hospital; d Department of Dermatology, Nagoya University Hospital; e Department of Dermatologic oncology, National Cancer Center Hospital; f Department of Dermatology, Tohoku University Graduate School of Medicine; g Department of Dermatology, Niigata Cancer Center Hospital; h Department of Dermatology, Wakayama Medical University; i Department of Preventive Medicine and Public Health, Keio University School of Medicine, Tokyo, Japan.

**Keywords:** epithelial skin malignancies, malignant cutaneous epithelial tumors, non-melanoma skin cancer, nivolumab, anti-PD-1 antibody

## Abstract

**Introduction:**

Malignant cutaneous epithelial tumors comprise various skin malignancies originating from the cutaneous epithelium, including cutaneous squamous cell carcinoma, basal cell carcinoma, and malignant cutaneous adnexal tumors. Treatment options are limited, as the rarity of these tumors, especially among Asians, renders well-controlled clinical trials extremely challenging to conduct. Thus, we designed a clinical trial to evaluate the efficacy and safety of the anti-programmed cell death-1 (PD-1) monoclonal antibody nivolumab in patients with metastatic cutaneous squamous cell carcinomas and other rare metastatic cutaneous epithelial tumors.

**Methods and analysis:**

This is an open-label, single-arm, multicenter, phase 2 clinical trial involving patients with metastatic malignant cutaneous epithelial tumors. Nivolumab (480 mg) will be administered intravenously every 4 weeks for a maximum of 26 doses. The primary outcome of the study will be the response rate based on response evaluation criteria in solid tumors, version 1.1. Assuming a null hypothesis of a response rate ≤5% and an alternative hypothesis of a 25% response rate, a minimum of 26 patients are required to achieve a 5% two-sided type I error and 80% power based on the exact binomial distribution. Finally, a target cohort size of 30 patients was determined as some patient dropout will be expected.

**Discussion:**

This is the first phase 2 clinical trial evaluating the efficacy and safety of the PD-1 inhibitor nivolumab in Asian patients with metastatic malignant cutaneous epithelial tumors. The findings of the study will contribute to the development of novel treatment approaches for patients with rare cutaneous malignancies, which remains an unmet clinical need.

**Trial registration:**

Registry number: jRCT 2031190048

## 1 Introduction

Malignant cutaneous epithelial tumors, also referred to as non-melanoma skin cancer (NMSC), are common human neoplasms arising from the epidermis or cutaneous appendages. These include basal cell carcinoma (BCC), cutaneous squamous cell carcinoma (cSCC), extramammary Pagets disease (EMPD), and several other skin adnexal carcinomas. Of these, BCC and cSCC are the most common forms, accounting for 75% to 80% and 20% to 25% of all NMSC cases, respectively; other types of are extremely rare.^[1,2]^ The incidence of those varies, depending on the race, skin type, and geographic region, with a lower incidence in Asians compared with Caucasians. In general, NMSC has a favorable prognosis after local resection of the primary lesion. Reported rates of metastasis range between 0.0028% and 0.55% for BCC, while that for cSCC is 2.5%.^[3,4]^ Skin adnexal carcinomas are also considered to have a low metastatic potential.^[5]^ However, patients with metastatic disease have an extremely poor prognosis.^[6–8]^ The median overall survival (OS) of BCC patients is 10.0 months (range, 0.5–108.0 months),^[6]^ whereas those of cSCC and EMPD patients are 2.19 and 1.5 years, respectively.^[7,8]^

Treatment of those advanced forms have long been done by the administration of cytotoxic chemotherapeutic agents. Patients with cSCCs are often treated with bleomycin, peplomycin, irinotecan, platinum, anthracycline, taxanes, or 5-fluorouracil.^[9–16]^ For EMPDs, single-agent taxane, cisplatin, or fluoropyrimidine-based regimens are used.^[17–25]^ Nevertheless, evidence regarding the clinical benefit of these therapies is limited, mainly due to the rarity of these cancers and the challenges involved in the design of well-controlled clinical trials. Conducting clinical studies in patients with adnexal carcinomas is even more challenging, as the incidence of adnexal carcinoma is extremely low. The majority of commonly used cytotoxic agents have not gained regulatory approval for the treatment of metastatic malignant cutaneous epithelial tumors.

Increasing knowledge regarding cancer pathobiology, as well as advances in molecular biotechnology, have accelerated the development of targeted therapies. As for cSCC, the efficacy and safety of the monoclonal anti-epidermal growth factor receptor antibodies cetuximab and panitumumab and the orally administrated small-molecule inhibitors gefitinib and erlotinib have been reported.^[26–29]^ Orally administered small-molecule inhibitors of the Hedgehog signal pathway, vismodegib and sonidegib, have been approved by the U.S. Food and Drug Administration (FDA) for the treatment of advanced BCCs.^[30–33]^ The anti-epidermal growth factor receptor 2 (HER2) humanized monoclonal antibody trastuzumab was reported to provide a clinical benefit in patients with HER2-positive EMPD.^[34–37]^

Monoclonal antibodies targeting immune checkpoint molecules have emerged as promising therapeutic approaches for several human cancers. Particularly, monoclonal antibodies targeting programmed cell death 1 (PD-1) and its ligand, programmed cell death ligand 1 (PD-L1), have revolutionized the landscape of malignant skin cancer.^[38]^ The PD-1/-L1 pathway plays a critical role in tumor immune evasion across a broad range of tumor types. PD-1 is highly expressed in activated lymphocytes, whereas PD-L1 is expressed in cancer and stromal cells; binding of PD-L1 to PD-1 suppresses antitumor immune responses.^[39]^ Thus far, the FDA has approved the PD-1/PD-L1 inhibitors nivolumab and pembrolizumab for melanoma, cemiplimab for cSCC, and pembrolizumab and avelumab for Merkel cell carcinoma. However, no PD-1/PD-L1 inhibitors have been approved for use in cSCC patients in Japan. Furthermore, none of these agents are currently approved for rare adnexal tumors anywhere in the world.

To address these unmet medical needs, especially in Japanese patients with metastatic malignant cutaneous epithelial tumors, we designed a clinical trial to evaluate the efficacy and safety of nivolumab in Japanese patients with metastatic cSCC and other rare malignant cutaneous epithelial tumors.

## 2 Methods/design

### 2.1 Objectives, trial design, and study setting

This study is a single-arm, open-label, multi-institutional phase 2 clinical trial aiming to assess the efficacy and safety of nivolumab in patients with advanced malignant cutaneous epithelial tumors. The recruitment of study participants started in July 2019 and will continue until a total of 30 participants are registered. The study will be conducted in 8 centers: Keio University Hospital, Tohoku University Hospital, Niigata Cancer Center Hospital, National Cancer Center Hospital, Nagoya University Hospital, Kyoto University Hospital, Wakayama Medical University Hospital, and National Hospital Organization Kyushu Cancer Center. All study participants are required to sign an informed consent form by the investigators. Nivolumab will be administrated every 4 weeks for up to 26 cycles; the treatment will continue for up to 2 years in patients benefiting from the treatment who show no evidence of disease progression.

### 2.2 Eligibility criteria

#### 2.2.1 Inclusion criteria

1. Age: 20 years or older.
2. Histologically confirmed incurable, advanced, or recurrent epithelial skin malignancies after surgical treatment and/or radiotherapy.
3. ≥1 measurable lesions based on RECIST 1.1
4. ECOG performance status 0-1.
5. Life expectancy ≥90 days
6. Women willing to use double contraception and agree not to breastfeed for at least 5 months after final administration.
  Men willing to use double contraception for at least 5 months after final administration.
7. Sufficient organ functions: all of the following conditions are fulfilled:
  - White blood cell ≥2000/mm^3^
  - Absolute neutrophil count ≥1500/mm^3^
  - Platelets ≥100,000/mm^3^
  - Hemoglobin ≥9.0 g/dl
  - AST and ALT ≤ 3times the upper limit of normal
  - Total bilirubin ≤ 2 times the upper limit of normal
  - Serum creatinine ≤ 1.5 mg/dl or creatinine clearance or eGFR ≥ 45 ml/minute.

Patients received an adequate explanation about the study and provided informed consent.

#### 2.2.2 Exclusion criteria

1. History of anaphylaxis to other antibody formulations, severe allergy, chronic or recurrent autoimmune disease, transplantation therapy, recent transient cerebral ischemic attack or cerebral vascular accident, recent thrombosis, or thromboembolism.
2. History of additional malignancy except for completely resected BCC, carcinoma *in situ*, intramucosal cancer, superficial bladder cancer, or other cancers that have not recurred for at least 5 years before enrollment.
3. History of pretreatment using anti-PD1/L1/L2, anti-CD137, anti-CTLA-4 inhibitor or any other antibody or drugs intended for T-cell regulation.
4. Active central nervous system (CNS) metastases
5. Uncontrolled tumor-associated pain
6. Current disease; active autoimmune disease, diverticulitis or symptomatic peptic ulcer disease, pericardial effusion, pleural effusion or ascites requiring sustained treatment, uncontrollable diabetes mellitus, systemic infection requiring treatment, interstitial lung diseases, pulmonary fibrosis, or unstabled radiation pneumonitis, uncontrollable or severe cardiovascular disease.
7. Recent following treatments within 28 days prior to the enrollment; systemic adrenocortical hormone, immunosuppressant, unapproved drugs, any antineoplastic agent, surgical adhesion of the pleura or pericardium, surgery under general anesthesia, radiotherapy.
8. Received surgery under local or topical anesthesia within 14 days prior to the enrollment.
9. Received any radiopharmaceuticals within 56 days prior to the enrolment.
10. Seropositive for HBsAg, HCV-Ab, or HIV-Ab.
11. Pregnant, breast-feeding or potentially pregnant.
12. History of any condition or therapy that might confound the results of this study.

#### 2.2.3 Ethics approval and consent to participate

This study is being conducted in accordance with the principles expressed in the Declaration of Helsinki, good clinical practice guidelines and SPIRIT (Standard Protocol Items: Recommendations for Interventional Trials) guidelines. The protocol version 1.0 was approved by the Institutional Review Board of Keio University Hospital in May 2019 and the institutional review board of the following participating center: Tohoku University Hospital, Niigata Cancer Center Hospital, National Cancer Center Hospital, Nagoya University Hospital, Kyoto University Hospital, Wakayama Medical University Hospital, and National Hospital Organization Kyushu Cancer Center. Written informed consent has been obtained from all enrolled participants.

#### 2.2.4 Endpoints

The primary outcome of this study will be the response rate based on RECIST, version 1.1 criteria. Secondary outcome measures include the best overall response rate, progression-free survival (PFS), OS, disease control rate, and adverse events graded using the National Cancer Institute Common Terminology Criteria for Adverse Events, version 4.0. For this study, PFS will be defined as the time from initiation of treatment in the clinical trial to disease progression or death from any cause, and OS will be defined as the time from initiation of treatment in the clinical trial to death from any cause. The response rate based on the immune RECIST will also be assessed as an exploratory outcome measure.

#### 2.2.5 Treatment methods

The trial schedule is composed of 3 periods the screening, treatment, and post-observation periods. The screening period starts from the date of consent acquisition to the day before the first administration of the study drug. During this screening period, inclusion/exclusion criteria for the study participants will be evaluated. During the treatment period, 480 mg nivolumab will be administered intravenously over 30 minutes, every 4 weeks. Administration of the study drug will be continued for up to 26 doses, with a maximum treatment period of 2 years. Study participants will be assessed for a tumor response every 2 treatment cycles by imaging. Any patients who meet the criteria for discontinuation of the treatment or who have completed the 2-year administration period will be assessed for a tumor response and move into the post-observation period. During the post-observation period, adverse events will be reported 28 days after the last dose of nivolumab. Subsequently, tumor lesions will be monitored every 8 weeks by imaging during the follow-up period. To determine OS, all subjects will be followed-up until death, withdrawal of consent, or the end of the study.

#### 2.2.6 Criteria for discontinuation of the treatment

The administration of nivolumab will be discontinued in patients who experience at least 1 of the following:

1. a radiographic complete response according to the response evaluation criteria in solid tumors (RECIST) version 1.1, with the exception of cases with a high risk of recurrence;
2. radiographic disease progression according to the RECIST, version 1.1 or apparent disease progression with clinical symptoms;
3. grade 3 or higher interstitial pneumonia;
4. grade 2 or higher eye pain or blurred vision, regarded as a response to the study drug, showing no improvement to ≤ grade 1 after appropriate treatment;
5. grade 3 or higher bronchospasm, diarrhea, colitis, neurotoxicity, hypersensitive reaction, infusion reaction, or uveitis, regarded as a response to the study drug;
6. grade 3 or higher thrombocytopenia for ≥5 days;
7. more than 10 weeks from the start of the previous treatment cycle;
8. the investigator or physician decides to discontinue administration of the drug.

#### 2.2.7 Concomitant medications

Medications specifically prohibited in the exclusion criteria will be not allowed during the ongoing trial. Patients are also prohibited from receiving the following therapies during the screening and treatment phase of the study:

- Any investigational medication other than the study drug.
- Any anticancer therapies including chemotherapy or biologic therapy other than the study medication, excluding the follows:
- Intravenous infusion of zoledronic acid hydrate, pamidronate disodium or subcutaneous injection of denosumab, started for bone metastases before enrollment in this study

Systemic steroid therapy can be combined when immune-related adverse events caused by the study drug are suspected.

#### 2.2.8 Data collection

Data quality control will be conducted by a combination of centralized (remote) and on-site monitoring, according to each procedures standardized protocols. Centralized monitoring will be conducted by the coordinating investigator, the coordinating office, and the data center conducting the data collection. The data center will verify the progress of the clinical trial, the visit of each patient, protocol compliance, and the occurrence of adverse events. A centralized monitoring report will be created by the data center and submitted to the coordinating investigator, who will ensure that the study is conducted properly and safely based on the centralized monitoring report and will take appropriate measures when necessary.

The on-site monitoring will ensure that the study is conducted in compliance with the protocol and good clinical practice guidelines, as well as assess the electronic case report forms. The data manager will check for inconsistencies. The quality assurance auditor will assess the quality of the study independently according to the quality assurance plan. Moreover, the auditor will assess that the study is conducted in compliance with the protocol and good clinical practice guidelines.

#### 2.2.9 Sample size calculation

Assuming a null hypothesis of a response rate of ≤5% and an alternative hypothesis of 25%, a minimum of 26 patients are required to achieve a 5% two-sided type I error and 80% power based on the exact binomial distribution. A target sample size of 30 was set to account for an expected patient dropout of 10%.

### 2.3 Statistical analysis

Statistical analyses and reporting will be conducted in accordance with the Consolidated Standards of Reporting Trials guidelines, with the primary analyses based on the intent-to-treat principle without imputing any missing observations. All efficacy analyses will be based primarily on the full dataset, which includes all patients who have received at least 1e dose of nivolumab and were in accordance with the study protocol. Safety analysis will be conducted using data from the safety analysis population. For baseline variables, summary statistics will be performed using frequencies and proportions for categorical data and means and standard deviations for continuous variables.

The primary endpoint for efficacy will the proportion of responders, considering a response threshold of 5% (H_0_, null hypothesis) and an expected response rate of 25% (H_1_, alternative hypothesis). The 95% confidence intervals will be calculated according to the binomial distribution of the response rate. The confidence interval limits will be assessed against the response threshold.

Subgroup analysis will be performed to evaluate safety and efficacy depending on the tumor subtype. All statistical analyses will be performed using SAS software version 9.4 (SAS Institute). All statistical analyses will be described in detail in the statistical analysis plan, which will be fixed prior to database lock.

### 2.4 Confidentiality

All study-related information will be stored securely on-site and identified by a coded number only to ensure participant confidentiality. All records that contain names or other personal identifiers will be stored separately from study records identified by code number. All local databases will be secured with password-protected access systems. Data generated by this trial will be considered confidential by the investigators, except for data to be included in publications.

### 2.5 Patient and public involvement

No patient involved.

## 3 Discussion

This is a single-arm clinical trial that involves the use of nivolumab in Japanese patients with metastatic cSCC or other rare malignant cutaneous epithelial tumors. The rationale of this study relies on the several previous clinical and basic evidences (Table 1). In a phase I study assessing the usefulness of the PD-1 inhibitor cemiplimab in patients with metastatic and locally advanced cSCC, an overall response rate of 50% was reported. In a phase II study, the overall response rate was 47.5% (28/59 cases), including 4 cases of complete remission. By integrating the results of these 2 studies, the response rate of metastatic cSCC to cemiplimab was 46.7% (35/75 cases).^[40]^ The clinical benefit of anti-PD-1 antibodies for rare malignant cutaneous epithelial tumors have also been reported by several case reports. Successful treatment with nivolumab of advanced BCC patients refractory to Hedgehog signaling inhibitors has been reported.^[41]^ Another case report demonstrated that treatment with pembrolizumab resulted in a partial response in a patient with cutaneous apocrine carcinoma.^[42]^ The tumor mutation burden has been associated with the neoantigen burden and response to anti-PD-1 treatment in melanoma.^[43]^ Importantly, cSCCs are reported to have a high mutation burden among various cancer types.^[44,45]^ Furthermore, some EMPD cases demonstrated a similar tumor mutation burden as that in SCC cases.^[46]^ Based on these clinical and biological observations, we hypothesize that nivolumab will also show a clinical benefit in multiple malignant cutaneous epithelial tumor types, regardless of any biological and pathological differences.

**Table 1:**
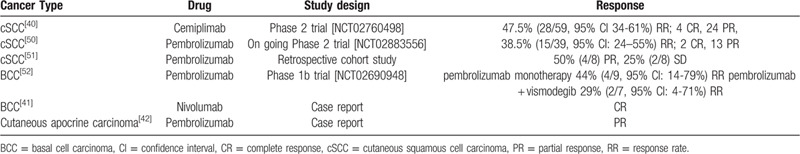
Reported anti-PD-1/-L1 therapies for advanced epithelial skin malignancies.

The fact that PD-1 inhibitors have gained regulatory approval for use in cSCC in the U.S. but not in Japan is mainly attributed to differences in the incidence of metastatic cSCCs. The incidence of cSCC in Caucasian populations is reported to be 17 to 360 per 100,000 cases, which is 5.8 to 138 times higher than that in Asian populations (2.6–2.9 per 100,000 cases).^[47]^ In a survey of the prevalence of advanced NMSCs in Japan, the annual reported number of advanced cSCC patients was 323.5.^[48]^ Nevertheless, the incidence of metastatic cSCC is increasing. The 5-year OS rate of cSCC patients treated with conventional cytotoxic chemotherapy is 26% to 29%, suggesting that metastatic cSCCs are also life-threatening among Japanese patients, and that the establishment of novel therapeutic approaches remains an unmet medical need.^[49]^ To address this issue, we designed this clinical trial involving Japanese patients with metastatic cSCC. We decided to also include patients with other metastatic adnexal tumors to potentially expand the range of cancer types treated with PD-1 inhibitors. Because these tumor types are extremely rare, well-controlled clinical trials involving patients with such tumors are unlikely to be conducted.

This study design has several limitations, most of which are because of the rarity of the tumor types involved. The first, number of patients enrolled will be low, and the cancer type varies among the study participants. The second, differences and heterogeneity in the response to nivolumab among the study participants are expected, due to potential differential sensitivity of the different cancer types to immunotherapy. Nevertheless, the present study could lead to the regulatory approval of nivolumab for use in Japanese patients with malignant cutaneous epithelial tumors, the treatment of which remains an unmet clinical need.

### 3.1 Trial status

The trial is currently actively recruiting patients. Completion of patient recruitment is expected for the beginning of 2021.

## Acknowledgments

We would like to thank the all members of study operation office and data center.

## Author contributions

MI, IH and KT contributed to the writing of the manuscript. T. Fun is the principal investigator and responsible for the organization and coordination of the trial. All authors contributed to the management and administration of the trial. T. Fun conceived the idea for the project and designed the study.

**Conceptualization:** Takeru Funakoshi.

**Funding acquisition:** Takeru Funakoshi.

**Methodology:** Yasunori Sato.

**Project administration:** Takeru Funakoshi.

**Supervision:** Masayuki Amagai, Takeru Funakoshi.

**Writing – original draft:** Maki Ishii, Ikuko Hirai, Keiji Tanase.

**Writing – review & editing:** Ikuko Hirai, Keiji Tanase, Takayuki Fusumae, Yoshio Nakamura, Keitaro Fukuda, Hiroshi Uchi, Kenji Kabashima, Atsushi Otsuka, Kenji Yokota, Naoya Yamazaki, Kenjiro Namikawa, Taku Fujimura, Tatsuya Takenouchi, Yuki Yamamoto, Mana Nishiguchi, Yasunori Sato, Masayuki Amagai.
